# “Doctor, I Hear Music”: A Brief Review About Musical Hallucinations

**DOI:** 10.2174/1874205X01711010011

**Published:** 2017-02-28

**Authors:** Purificacion Alvarez Perez, Maria Jose Garcia-Antelo, Eduardo Rubio-Nazabal

**Affiliations:** 1 Degree in Medicine and Surgery, Centro de atención primaria (CAP) Ventorrillo, A Coruña. Spain.; 2 Degree in Medicine and Surgery, Servicio de Neurología (Department of Neurology). Hospital Universitario A Coruña. A Coruña. Spain.

**Keywords:** Musical hallucinations, Complex auditory hallucinations, Auditory Charles Bonnet syndrome, Music, Dementia, Sound

## Abstract

Auditory hallucinations are defined as the abnormal perception of sound in the absence of an external auditory stimulus. Musical hallucinations constitute a complex type of auditory hallucination characterized by perception of melodies, music, or songs. Musical hallucinations are infrequent and have been described in 0.16% of a general hospital population. The auditory hallucinations are popularly associated with psychiatric disorders or degenerative neurological diseases but there may be other causes in which the patient evolves favorably with treatment. With this clinical case we want to stress the importance of knowing the causes of musical hallucinations due to the unpredictable social consequences that they can have.

## INTRODUCTION

 An auditory hallucination is defined as an abnormal perception of sound in the absence of an external auditory stimulus. Auditory hallucinations can be simple (unspecific sound, in the form of tinnitus...) or complex (more elaborate, of a more complex content). Musical Hallucination (MH) constitutes a complex type of auditory hallucination in which the perception of said hallucination can be in the form of melodies, music or songs.

As figures in the scientific literature, Emmanuel Régis (a French psychiatrist), was the first to mention musical hallucinations (in 1881). But it was not until some 50 years later, in 1932, when the difference between musical hallucinations of organic or psychotic origins was established. Since then sets of cases have been published and some studies, in spite of which the prevalence of musical hallucinations cannot be clearly defined; it is believed that approximately 0.16% of the population of a general hospital [1].

The MH can be of different origin. They have been linked to psychiatric illnesses such as the cases of schizophrenia, depression or obsessive-compulsive disorders. However, itis not in the psychiatric environment where they are more prevalent. The organic MH are prevalent above all in patients with neurological pathologies, whether these be cerebral lesions (vascular cerebral diseases, tumors, demyelinating disease...), epilepsy of the temporal lobe, Parkinson's, infectious processes of the central nervous system, etc. They are also produced in patients with severe hearing loss constituting the auditory Charles Bonnet Syndrome (CBS). They can be produced as a side effect of some medications (pentoxifylline, tramadol, Bromocriptina) and have also been observed in hepatic transplants. And sometimes it's difficult to know its etiology [2].

There is no specific treatment; the majority of cases in which treatment has been effective depended on the resolution of the underlying cause. We present the case of a woman who came in for a consult due to musical hallucinations in which the correct diagnosis and treatment allow the patient to have personal autonomy avoiding institutionalization for her condition.

## CASE REPORT

81-year-old woman with a history of hypertension treated via Enalapril and dyslipidemia treated via diet. She had been evaluated some years ago by an otorhinolaryngologist and was diagnosed with bilateral hypoacusis, which was severe in the right ear and moderate in the left ear, where she had a hearing aid.

Four months prior to the consult, the patient had her first episode of MH. It consisted of childhood songs, which she had learned and liked (Manolo Escobar, folk songs). She heard them in her left ear, which was the only one that still retained partial audition with a hearing aid. She had not heard anything with her right ear for several years. The songs could last for hours and did not bother her; she assumed they came from the outside environment.

It was noted that the patient did not have any previous incidents of psychiatric illness, she had not shown symptoms of cognitive deterioration, and she had no changes in her regular treatment; she lived alone and was independent in her daily life. After the patient’s symptoms began, her family was worried about with the possibility that she had a psychiatric or neurological illness; following a consult with her general practitioner the patient was given treatment with sertraline. The family also started considering the possibility of sending her to a nursing home believing that she was showing signs of the onset of dementia.

In the neurological examination, aside from the hypoacusis, the patient did not present with alterations of interest and, in her cognitive evaluation, the tests were within normal ranges (Minimental-test, Fototest).

Because of the presence of MH, the patient was evaluated by psychiatry, which did not encounter any pathology.

In the tests performed: analytical, Holter EEG, and MRI of her brain, only diffuse cerebral atrophy was identified in the image study, and there were no other points of interest. The audiometry test noted a moderate loss of neurosensory hearing in the left ear and severe loss in the right.

Once diagnosed with auditory hallucinations secondary to sensory deprivation, she was placed on carbamazepine (200 mg every 8 hours). About 15 days later there was a partial improvement of the clinic: the music came less frequently, although of equal intensity. After explaining the symptoms to the patient and her family, she continued living alone and having personal autonomy

## DISCUSSION

Musical hallucinations are an infrequent type of auditory hallucination. MH can be simple or complex. Simple MH (tinnitus), includes a wide range of sounds (whistling, hissing, squealing, ringing…). Complex musical hallucinations refer to more elaborate sounds as music or songs or speech and those are the ones we will discuss in this article [3].

There are various risk factors associated with the presence of MH (Table **1**): advanced age, female sex (up to 70-80% in some cases), presence of auditory deficit, social isolation, treatment with certain pharmaceuticals, presence of cerebral atrophy in image studies, and psychiatric pathology [4].

In relation to the etiology (Table **2**), MH are divided into five groups: secondary to auditory deprivation [5], focal cerebral lesions [4, 6, 7], psychiatric pathology [3], epilepsy [4], and secondary to pharmaceuticals or metabotoxic causes [8]. Auditory deprivation as a cause of MH is observed in older patients with moderate or severe loss of hearing and the MH are lateralized to the ear in which audition is partially mantained. In general, patients with MH perceive, above all, familiar sounds (melodies learned in their childhood) and the content of the MH is more frequently voices combined with instruments than purely musical; also, MH more frequently elicits a negative emotional reaction rather than a pleasant or neutral one. In the case of auditory deprivation, MH can be interpreted as a phenomenon of deafferentation, as if we were describing an auditory Charles-Bonnet Syndrome [9]. The physiopathological mechanism of MH is unknown, but it is known that it is associated with learned melodies, which suggests that MH are derived from perceptual experiences accumulated in musical memory circuits. In cases of deafness or compromise of the pontine inhibitory pathways over these musical memory circuits, an anomalous disinhibition of these circuits and liberation of this type of memory in the form of MH would be produced [6, 7]. In this sense, Schielke et al. revise various cases of lesions affecting the brainstem as a cause of MH (hemorrhagic and ischemic lesions, encephalitis, abscesses). In these cases, MH appear between the first and fourteenth day after the lesion, disappearing in many cases in the ensuing weeks and months. The most frequent psychiatric pathology associated with MH is depression, schizophrenia and obsessive-compulsive disorder although musical hallucinations are uncommon in psychiatry. Musical hallucinations have been commonly reported among patients with epilepsy, but it is rare that epilepsy starts with MH alone; usually there are other accompanying symptoms that guide the diagnosis [10]. They are mostly associated with temporal lobe lesions. MH secondary to pharmaceuticals have been fundamentally associated with antihypertensives, but there are isolated cases secondary to other pharmaceuticals (propranolol, imipramine, tramadol, salicylates, pentoxifylline, amitriptyline, triazolam, clomipramine, marijuana, benzodiazepines, amphetamines, quinine, phenothiazine, carbamazepine, paracetamol, phenytoin, procaine, alcohol, and general anesthetics). The metabotoxic causes that have been described are Hashimoto’s encephalopathy and Lyme disease [3, 4].

Treatment [12, 13] for the symptoms of MH is not known. There is no definitive treatment for musical hallucinations. Treatment is aimed to treat the underlying cause if it is known. The majority of cases in which treatment has been effective depended on the resolution of the underlying cause (improving auditory deprivation, suspending the responsible pharmaceutical…). Isolated cases have been described in which symptoms improved with: neuroleptics (quetiapine, olanzapine), antidepressants (fluvoxamine, clomipramine), donepezil, and antiepileptics (carbamazepine, valproate). One third of patients with auditory deprivation show depressive symptoms, so in these cases antidepressants have a greater benefit than neuroleptics [2, 3, 11]. Some authors suggest that an increase in external auditory stimulation can reduce the severity of persistent MH.

## CONCLUSION

In this case we want to stress the importance of knowing the causes of MH and reassure the patients and family, as hallucinations are popularly associated with psychiatric disorders or degenerative neurological diseases. A misdiagnosis and incorrect treatment can, on the one hand, distress the patients and their family, and on the other, have unpredictable social consequences.

## Figures and Tables

**Table 1:** Risk factors of musical hallucinations.

**RISK FACTORS OF MUSICAL HALLUCINATIONS**
Elderly (mean age> 61 years)
Sex: female (70-80% of cases)
Hearing loss
Drugs (primarily antihypertensives)
Brain disease (cerebral atrophy)
Brain disease (cerebral atrophy)
Social isolation

**Table 2:** Etiology of musical hallucinations.

**ETIOLOGY OF MUSICAL HALLUCINATIONS**	
Auditory Deprivation	
Psychiatric Disorders	Depression Schizophrenia Obsessive compulsive disorder
Focal brain lesions and cerebral atrophy	Ischemic and hemorrhagic stroke Tumors Multiple Sclerosis Rhomboencephalitis Calcifications of the basal ganglia Schizencephaly
Epilepsy	
Drugs	Hypotensive drugs Tricyclic antidepressants Antimalarials Other: Amantadina, baclofeno, salicylates, tramadol, pentoxifylline, triazolam, marijuana, benzodiazepines, amphetamines, phenothiazine, carbamazepine, paracetamol, phenytoin, procaine, pramipexol, topiramato, alcohol, and general anesthetics
Toxic-metabolic causes	Hashimoto's disease Lyme disease

## LIST OF ABBREVIATIONS

EEG: = electroencephalogram
MH: = musical hallucinations
MRI: = magnetic resonance imaging
